# The Microbial Landscape of Sea Stars and the Anatomical and Interspecies Variability of Their Microbiome

**DOI:** 10.3389/fmicb.2018.01829

**Published:** 2018-08-13

**Authors:** Elliot W. Jackson, Charles Pepe-Ranney, Spencer J. Debenport, Daniel H. Buckley, Ian Hewson

**Affiliations:** ^1^Department of Microbiology, Cornell University, Ithaca, NY, United States; ^2^AgBiome, Inc., Research Triangle Park, NC, United States; ^3^Indigo Agriculture, Boston, MA, United States; ^4^School of Integrative Plant Science, Cornell University, Ithaca, NY, United States

**Keywords:** microbiome, sea star, echinoderms, 16S rRNA, DADA2, Asteroidea, invertebrate

## Abstract

Sea stars are among the most important predators in benthic ecosystems worldwide which is partly attributed to their unique gastrointestinal features and feeding behaviors. Despite their ecological importance, the microbiome of these animals and its influence on adult host health and development largely remains unknown. To begin to understand such interactions we sought to understand what bacteria are associated with these animals, how the microbiome is partitioned across regions of the body and how seawater influences their microbiome. We analyzed the microbiome composition of a geographically and taxonomically diverse set of sea star taxa by using 16S rRNA gene amplicon sequencing and compared microorganisms associated with different regions of their body and to their local environment. In addition, we estimated the bacterial and coelomocyte abundance in the sea star coelomic fluid and bacterioplankton abundance in the surrounding seawater via epifluorescence microscopy. The average bacterial cell abundance observed in the coelomic fluid was one to two orders of magnitude lower than the bacterioplankton abundance in the surrounding seawater suggesting a selection against the presence of microorganisms in the coelomic fluid. The sea star microbiome was also significantly different from seawater with relatively few shared microbial taxa. Microbial communities were found to be significantly different between the pyloric caeca, gonads, coelomic fluid, and body wall of the animals. The most noticeable difference between anatomical sites was the greater relative abundance of *Spirochaetae* and *Tenericutes* found in hard tissues (gonads, pyloric caeca, and body wall) than in the coelomic fluid. The microbiome of sea stars thus appears to be anatomically partitioned, distinct from the microbial community of seawater and contains a relatively low abundance of bacteria within the coelomic cavity.

## Introduction

Sea stars, like all echinoderms, are strictly marine organisms that are found globally in a variety of different benthic environments including the rocky intertidal, coral reefs, abyssal plains, and polar waters where they commonly occupy the top trophic level as predators. In rocky intertidal environments sea stars, notably *Pisaster ochraceus* and *Stichaster australis*, can have dramatic impacts of the community composition through the regulation of mussels and have been labeled as keystone species meaning they have a disproportionate impact relative to their abundance (Paine, 1966, 1971, 1974). Although sea stars typically act as carnivores that preferably prey on sessile or free-moving living animals, sea stars are opportunistic feeders that will scavenge on decaying animal material or feed on organic film substrates (Jangoux, 1982). Few sea star taxa specialize on specific prey, and their ability to consume a wide range of organic material is attributed to their feeding behavior and gastrointestinal features. Despite their ecological importance, the sea star microbiome largely remains uncharacterized and the role microorganisms may have on sea star physiology remains unknown.

Microorganisms associated with metazoa have profound impacts on host health and development by altering host behavior, immunity, digestion, and reproduction (Gil-Turnes et al., 1989; Engelstädter and Hurst, 2009; Hadfield, 2011; Shin et al., 2011). These impacts can be mediated by individual microorganisms or by complex communities through a wide range of mechanisms and can differ in a host-tissue specific manner. A common question in microbiome research is whether different organs or body regions within a host differ in microbial community composition. In vertebrates the microbiome is highly partitioned across the body (e.g., gastrointestinal tract, skin, urogenital cavities, and oral cavity) reflecting varying environmental conditions suitable for different microbial taxa. Invertebrates, however, often lack many of these differentiated body sites so it is not clear how the microbial landscape is shaped across this diverse group of animals. Among the most well studied non-human systems for animal symbiosis are marine invertebrates that includes the Hawaiian bobtail squid (*Euprymna scolopes*) (Nyholm and McFall-Ngai, 2004), corals (Baker, 2003), shipworms (Mollusca: Teredinidae) (Distel et al., 2002), sponges (Hentschel et al., 2012), and vestimentiferan tubeworms (Dubilier et al., 2008). The complexity and spatial organization of the microbiota in these examples differs for each animal.

**FIGURE 1:**
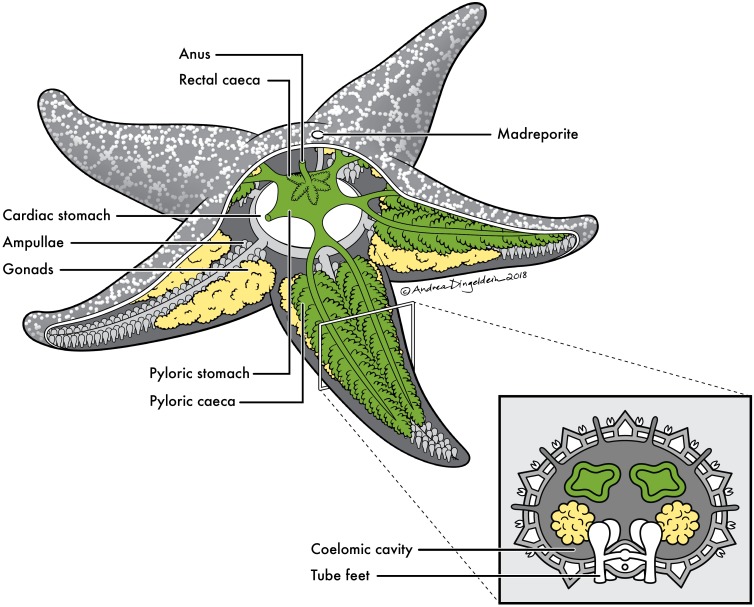
Sea star anatomy. Basic anatomy of a sea star with cross section of a ray. Anatomical sites sampled for this study include: (1) pyloric caeca (green), (2) gonads (yellow), (3) body wall (gray) and (4) coelomic fluid contained within coelomic cavity.

In this study we investigated the microbial communities of sea stars across four regions of the animal, between host taxa, and compared these communities to their local environment to understand how the microbiome of these organisms are shaped. We examined the sea star microbiome using high throughput 16S rRNA gene amplicon sequencing of the V4 region to compare the microbiomes of 12 sea star taxa from two contrasting habitats (coral reef vs rocky sub-tidal) and across four different regions within the animal (body wall, gonads, pyloric caeca, and coelomic fluid) (**Figure 1**). We found the sea star microbiome to be significantly different between anatomical regions of the animal, animals collected from different geographic locations, and significantly different from the microbial community in seawater. We also found up to a two order of magnitude reduction of bacterial cells in the coelomic fluid of sea stars compared to bacterioplankton in the surrounding seawater.

## Materials and Methods

### Sample Collection

Samples used in this study were collected from various locations in the Salish Sea off the coast of Washington State, United States and two locations off the coast of Queensland, Australia (**Supplementary Table 1**). Sea stars used in this study were photographed for taxonomic verification. Animals collected from the Salish Sea had no symptoms of Sea Star Wasting Disease. Sea stars in the Puget Sound were collected onboard the R/V Clifford Barnes by dredging from depths between 24 and 50 m (**Supplementary Table 1**). Surface water samples were taken from the same locations where sea stars were collected. The Puget Sound is a highly mixed body of water that experiences little stratification during the winter period. Because of this, the microbial community of surface waters would likely not be very different from the depth the animals were collected (Moore et al., 2008). Twenty liters of surface water were collected and prefiltered through a 150 mm GF/A [Cat.No 1820-150] then collected on a 142 mm, 0.22 μm Durapore membrane filter [Cat. No GVWP14250] (**Supplementary Table 1**). Sea stars collected in Queensland, Australia were from Moreton Bay and Heron Island, collected during low tide at one to two meters depth (**Supplementary Table 1**). One to two liters of surface water was collected at sites of animal collection by filtering through a 0.22 μm sterivex filter [Cat. No SVGP01015] (**Supplementary Table 1**). Sea stars were processed immediately upon collection. Coelomic fluid was extracted first using a 25G × 1½ (0.5 mm × 25 mm) needle [Cat. No 305127] attached to a 3 mL syringe [Cat. No 309657] inserted through the body wall into the coelomic cavity. Pyloric caeca and gonads were collected from the coelomic cavity using sterile forceps after making a small incision into the body wall. Tube feet and body wall tissue along the ambulacral groove were collected by vivisection. All samples were frozen in liquid nitrogen upon collection in sterile 15 mL tubes and kept at −80°C until processing. Tissue and fluid samples from three adult *Evasterias troschelii* originally collected from Dutch Harbor, Alaska but kept at Cornell University in aquaria containing artificial seawater were also sampled. A total of 106 samples were collected and processed for sequencing.

### DNA Extraction and Sequencing

DNA extractions were performed following the manufacturer protocols for each sample using Zymo Research Fungal/Bacterial DNA Miniprep kits [Cat. No D6005]. Roughly ¼ of the 142 mm, 0.22 μm Durapore membrane filter was used for DNA extraction while the whole sterivex filter was used for DNA extraction. Approximately 100 mg of animal tissue and 1–2 mL of coelomic fluid were used for DNA extraction. The coelomic fluid was first spun at 15,000 × *g* for 5 min then resuspended in 200 μl of nuclease-free water. Following DNA extraction, samples were held at −20°C prior to PCR amplification.

PCR reactions were carried out in 96-well plates using dual-indexed barcoded primers of the V4 region of the 16S rRNA gene (Caporaso et al., 2011; Kozich et al., 2013). For each sample 50 ng of DNA template was amplified in triplicate 25 μl total volume PCR reactions using the 515f (5′ – GTGCCAGCMGCCGCGGTAA – 3′) and 806r (5′ – GGACTACHVGGGTWTCTAAT – 3′) primers at 50°C annealing temperature for 30 cycles. Triplicate samples were pooled after PCR amplification, purified with SequalPrep^TM^ Normalization Plate (ThermoFisher Scientific) and quantified via PicoGreen (Invitrogen, Quant-iT^TM^ PicoGreen^®^ dsDNA Assay Kit). All amplicon products were pooled in equal concentrations for sequencing using Illumina MiSeq 500 bp sequencing v2 ^∗^ (2 × 250 bp) at the Cornell Institute of Biotechnology. Triplicate blanks consisting of elution solution as template were performed with each PCR run but no amplification was observed. The elution solution used for blanks however were not run through the Zymo DNA extraction kits.

### Read Quality Control and Analysis

Reads were first demultiplexed then analyzed for quality to determine trimming parameters. Reads were inspected by the average quality per base of the forward and reverse reads separately. The first 10 nucleotides for each read was trimmed and the total length of reads were truncated to 150 nucleotides due to the decrease in quality score observed after 150 nucleotides in both the forward and reverse reads. Reads containing any ambiguities were removed as were reads exceeding the probabilistic estimated error of 2 nucleotides. Quality parameters were enforced on both paired-end reads and if one of the reads did not pass the filtering parameters both reads were removed. After quality screening and trimming, the DADA2 pipeline was used to remove chimeric variants and to identify sub-OTUs (Callahan et al., 2016). Sub-OTUs are defined by analysis of polymorphic sites within amplicons and have been shown to have a greater taxonomic sensitivity than OTUs clustered by a 3% dissimilarity threshold (Callahan et al., 2016; Thompson et al., 2017). Analysis of sub-OTUs in place of OTUs has proven effective in resolving fine scale ecological temporal dynamics and community changes in the human microbiome which is why we used sub-OTUs rather than OTUs in analyzing the sea star microbiome (Eren et al., 2014; Tikhonov et al., 2015). The SILVA 123 database was used for taxonomic assignment. Reference sequences in the SILVA 123 database were first trimmed to the V4 region with the 515f-806r primers used in the PCR. Taxonomy assignments were performed using UCLUST with a minimum confidence threshold of 80% (Edgar, 2010). Sequences identified as chloroplast or mitochondria were removed from libraries prior to analysis. The relative abundance of chloroplast and mitochondrial reads across all libraries was <0.000001% which had a minimal effect on library size upon removal. Diversity (α and β), community composition, and statistical analyses were performed using the phyloseq R package and the vegan R package (Oksanen et al., 2007; McMurdie and Holmes, 2013). Only libraries containing at least 1,000 reads were used for analysis, and sub-OTU relative abundance values were calculated by transformation to library read depth. In total 86 libraries were analyzed (**Supplementary Table 1**). Unweighted Unifrac dissimilarity values were used for β-diversity measurements (Lozupone and Knight, 2005). Bray-Curtis dissimilarity values were also used for β-diversity measurements to complement Unifrac analysis. Principal Component Analysis (PCoA) were used to visualize β diversity, and the significance of grouping variables (collection location, sample type, host vs environmental) were assessed using Adonis test (Oksanen et al., 2007). Finally, LEfSe (Linear discriminant analysis effect size) was used to identify sub-OTUs significantly different among body regions of the animal (Segata et al., 2011). All of the script generated to analyze the data can be found at https://github.com/ewj34/Sea-Star-Microbiome.

### Enumeration of Bacteria in Coelomic Fluid

Coelomic fluid samples were prepared from four sea star species (*n* = 20) housed in artificial seawater in aquaria at Cornell, originally collected from Alaska and Washington, and one species (*n* = 5) in the field near Santa Cruz, CA, United States. Sea star species housed in aquaria included *Pisaster ochraceus* (*n* = 9)*, Pisaster brevispinus* (*n* = 5)*, Solaster stimpsoni* (*n* = 3), and *Evasterias troschelii* (*n* = 3). The sea star species sampled in the field was *Pisaster ochraceus* (*n* = 5). Artificial seawater used in aquaria at Cornell was maintained at a constant temperature of 53–54°F and a salinity of 34-35‰. Forty to 50 μl of coelomic fluid was extracted from sea stars as described above for epifluorescence microscopy (Patel et al., 2007). One mL of aquarium seawater (*n* = 5) and 0.5 mL of tide pool seawater (*n* = 3) where the animals were housed or sampled was also collected for epifluorescence microscopy. Seawater and sea star samples collected in the field were preserved in 2% formalin and processed 2 days later; samples taken from animals in aquarium were processed immediately without any preservation. Coelomic fluid samples were first added to 440–450 μl of 0.01% Tween 20 + 0.02 μm filtered 1X PBS solution. Two blank slides were prepared using 500 μl of 0.01% Tween 20, 0.02 μm filtered 1X PBS solution. Samples were then filtered in a glass housing unit through a 0.2 μm 25 mm anodisc filter [Cat. No 6809 6022] using a 0.8 μm, 25 mm backing filter [Cat. No AAWP02500]. SYBR gold was used to stain the anodisc filters. A 100x fluorescence oil-immersion objective with immersion oil was used to view the slide containing the filters. A 10 × 10 optical micrometer was used for counting cells. Bacterial cells were counted in 20 fields of view per sample. The abundance of bacteria (cells ml^−1^) was calculated by multiplying the average count per field by the total fields per filtration area and divided by the volume filtered.

## Results

**FIGURE 2:**
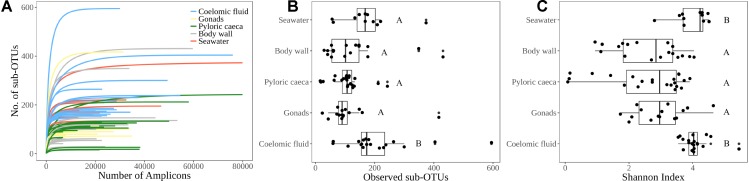
α-diversity indices. **(A)** Rarefaction curve for each sample library colored by sample type indicating sufficient sequencing depth for estimated microbial richness. **(B)** Observed richness of sub-OTUs grouped by sample type **(C)** Shannon diversity index grouped by sample type. Lettering corresponds to significantly different groups (*p* < 0.05) using Wilcoxon pairwise rank sum test with Bonferroni correction.

We analyzed the α diversity, β diversity, and community composition of *Bacteria* and *Archaea* for 75 sea star samples and 11 seawater samples (**Supplementary Table 1**). The 75 sea star samples were cataloged by sample type that included 19 coelomic fluid, 15 gonads, 22 pyloric caeca, and 19 body wall samples (**Supplementary Table 1**). The 75 sea star samples were taken from 12 sea star taxa (**Supplementary Table 1**). The sum of reads for all libraries passing the quality control parameters for this study totaled 2,229,468 reads with a mean library depth of 25,924 reads/library. The read depth of the libraries was sufficient to capture the total richness in the samples as all libraries reached saturation in the rarefaction curve (**Figure 2A**).

### α Diversity

**FIGURE 3:**
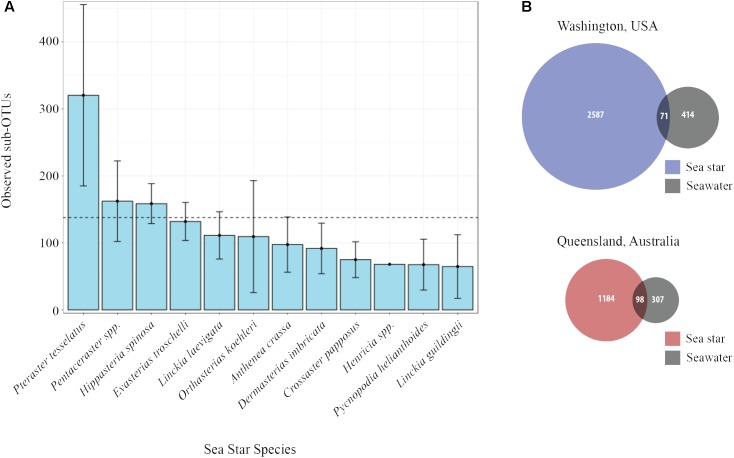
Sub-OTU richness among and between sea stars and seawater. **(A)** Observed richness of sub-OTUs among sea star species. Error bars represent standard errors. The dashed line is set at 137 and is the average number of sub-OTUs found across all sea stars. **(B)** Venn diagram of shared and unique sub-OTU between sea stars and seawater.

The average number of sub-OTUs found among host taxa ranged from 64 – 322 with an overall average of 137 sub-OTUs found among sea stars (**Figure 3A**). In total 2587 and 1184 unique sub-OTUs were found among sea stars from Washington, United States and Queensland, Australia respectively. Fewer than 10% of sub-OTUs associated with sea stars were found in seawater (**Figure 3B**). Richness between sample types was significantly different (Kruskal–Wallis, χ^2^= 20.68, *p* = 3.66 × 10^−4^) with fewer sub-OTUs observed in the pyloric caeca (107 ± 11; mean ± SE), gonads (112 ± 23), and body wall (124 ± 24) than in the coelomic fluid (209 ± 28) and seawater (173 ± 26) (**Figure 2B**). Likewise, Shannon diversity differed significantly between sample types (Kruskal–Wallis, χ^2^= 42.796, *p* = 1.14 × 10^−8^) with lower diversity in gonads (2.96 ± 0.79; mean ± SE), pyloric caeca (2.52 ± 0.27), and body wall (2.64 ± 0.23) than in coelomic fluid (4.08 ± 0.1) and seawater (3.97 ± 0.16) (**Figure 2C**). No significant difference was found between the Shannon diversity of seawater and coelomic fluid (Wilcoxon Rank-Sum Test, *p* = 1.00) though the coelomic fluid was significantly different from other sea star body sites (Wilcoxon Rank-Sum Test, *p* < 0.05).

### β Diversity

**FIGURE 4:**
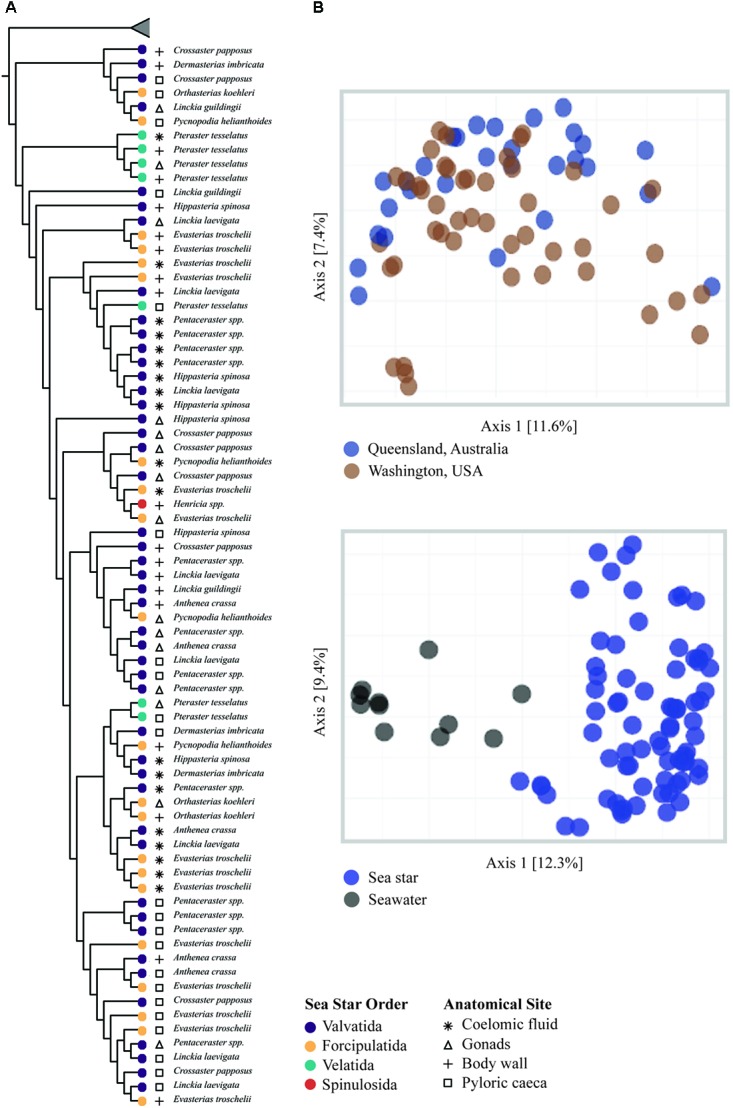
β-diversity analysis of sea star and seawater microbial communities. **(A)** UPGMA clustering based on unweighted Unifrac distances. Node tips are labeled by sea star taxa and node colors correspond to the respective order. Symbols correspond to sample type. Collapsed samples represent seawater samples. **(B)** PCoA plots generated from unweighted Unifrac distances.

All grouping variables (geographic location, sample type) were found to be significant (**Figure 4**, **Table 1**, and **Supplementary Figure 1**). *β* diversity analysis conducted using Bray-Curtis distance metrics produced similar results to unweighted Unifrac (**Table 1** and **Supplementary Figure 1**). LEfSe analysis using sample type as a grouping factor did not identify any sub-OTUs that were statistically different by relative abundance between the four body regions.

**Table 1:** Adonis results.

	Unweighted UniFrac			Bray-Curtis		
Partition Variable	F.Model	*R*^2^	Pr(>F)	F.Model	*R*^2^	Pr(>F)
Sea star and seawater	9.26	0.1	0.001^∗^	15.09	0.15	0.001^∗^
Sample type	1.74	0.07	0.001^∗^	3.98	0.15	0.001^∗^
Collection location	2.25	0.03	0.001^∗^	2.86	0.04	0.003^∗^

*Permutational multivariate analysis of variance (PERMANOVA) using unweighted Unifrac and Bray-Curtis distance metrics partitioned by various grouping variables. ^∗^Indicates p-value < 0.05.*

### Community Composition

**FIGURE 5:**
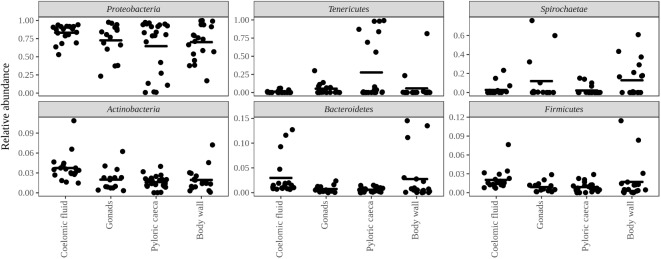
Top six bacterial phyla associated with sea stars. Top six bacterial phyla categorized by sample type with points representing individual sample libraries. Horizontal black bar represents the mean value for the phyla associated with the respective sample type.

**FIGURE 6:**
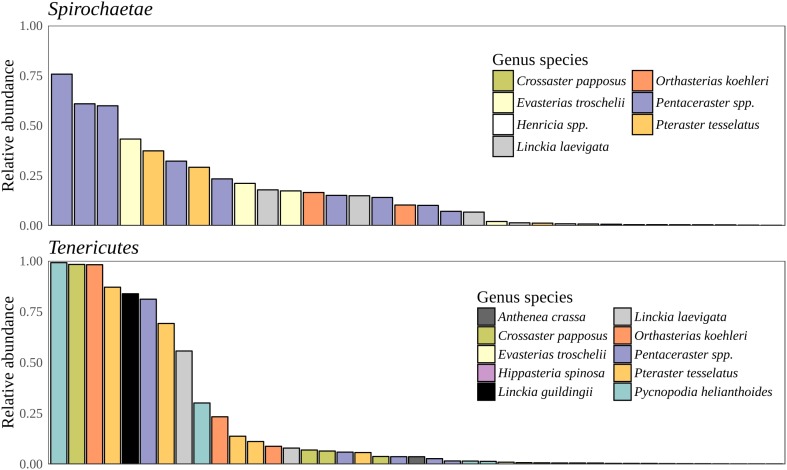
*Spirochaetae* and *Tenericutes* relative abundance. Total relative abundance of *Spirochaetae* and *Tenericutes* sub-OTUs in libraries. Colors correspond to sea star taxa.

*Bacteria* constituted 97% of sub-OTUs observed in the sea star microbiome, with *Archaea* and unassigned sequences comprising the remaining 3%. Six bacterial comprised 96% of the community composition: *Actinobacteria, Bacteroides, Firmicutes, Proteobacteria, Spirochaeata*, and *Tenericutes*. *Proteobacteria* consistently dominated the community making up an average of 73% of the sea star microbiome (**Figure 5**). The relative abun-dance of *Alpha-, Beta*-, and *Gammaproteobacteria* were 27, 21, and 23% respectively and collectively comprised the majority of *Proteobacteria*. *Deltaproteobacteria* made up 2% of the relative abundance of *Proteobacteria* and *Epsilonproteobacteria* made up <1%. The largest differences among the sea star sample types occurred in relation to the *Tenericutes* and *Spirochaetae* which were found in the hard tissues (i.e., pyloric caeca, body wall, and gonads) (**Figures 5**, **6**). *Tenericutes* had the second highest average relative abundance across all libraries (11%) but were highly variable among sample types, ranging from 1% in the coelomic fluid to 28% in the pyloric caeca. *Tenericutes* were present at unusually high relative abundance compared to their average across all libraries (>69%) in 7 of the 75 sea star samples (6 pyloric caeca and 1 body wall) (**Figure 6**). If these samples are removed from the analysis, the total relative abundance of *Tenericutes* across sea star libraries drops from 10.88 to 2.9%. *Spirochaetae* had the third highest average relative abundance (6.97%) across samples but was higher on average in the gonads and body wall compared to the coelomic fluid and pyloric caeca (12–13% to 1–2% respectively) (**Figure 5**). The samples with the top two highest relative abundance of *Spirochaetae* came from the body wall and gonads sampled from a single individual (*Pentaceraster* spp.) (**Figure 6**). *Actinobacteria, Bacteroides*, and *Firmicutes* were consistently found across all libraries but made up a small relative abundance collectively (<6%) (**Figure 5**).

**FIGURE 7:**
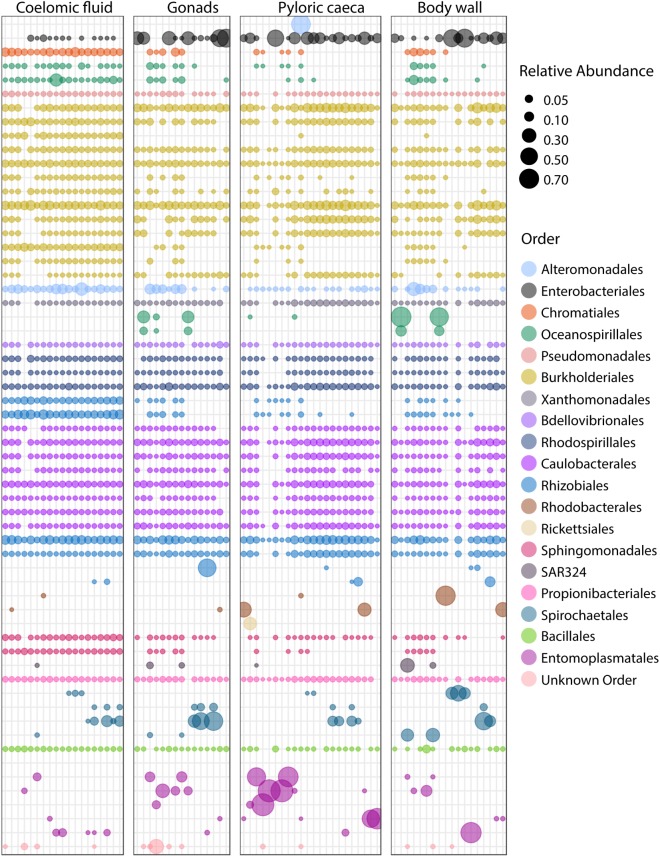
Bubble plot of top 60 sub-OTUs. Top 60 sub-OTUs are defined by mean relative abundance across all sea star libraries. Each column is a unique library. Color of bubbles correspond to taxonomic order of sub-OTU, and bubble size corresponds to relative abundance of a sub-OTU in a library.

We selected the top sub-OTUs among the sea star samples to characterize the microbial composition at a finer taxonomic level and compare these top taxa among sample types. We defined this set by rank order of the mean relative abundance for all sub-OTUs in sea star libraries. This set of sub-OTUs included 60 sub-OTUs in 20 taxonomic orders that made up 80% of all reads. (**Figure 7**). Sub-OTUs in *Propionibacteriales, Bacillales, Sphingomonadales, Rhodospirillales, Rhizobiales, Caulobacterales, Bdellovibrionales, Enterobacteriales, Alteromonodales, Xanthomonadales, Entero-bacteriales, Pseudomonodales*, and *Burkholderiales* were found in nearly all samples (**Figure 7**).

### Bacterial Cell Abundance

**FIGURE 8:**
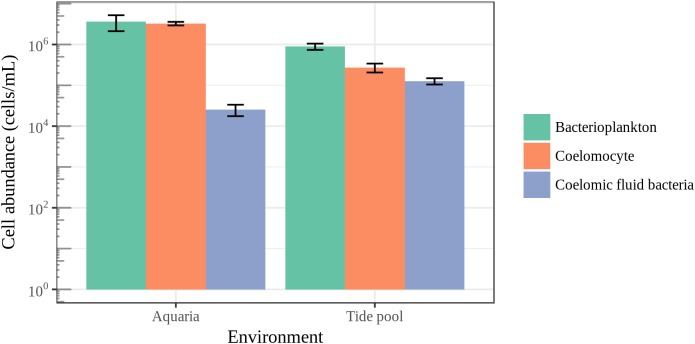
Epifluorescence microscopy cell counts. Enumeration of coelomocytes and bacterial cells in the coelomic fluid of sea stars and bacterioplankton in the surrounding seawater. Error bars represent standard error.

To put microbial diversity measures and community composition analyses into a meaningful context with regards to the host biology, we measured coelomocyte and bacterial cell abundance in the coelomic fluid and bacterial cell abundance in their surrounding seawater (**Figure 8**). The average bacterial cell abundance in the coelomic fluid was found to be two orders of magnitude lower than the average abundance of host cells and bacterioplankton for animals housed in aquarium. For animals housed in aquaria, bacterial cell abundance in the coelomic fluid was 2.55 × 10^4^ ± SE 7.90 × 10^3^ cells/mL. Coelomocyte abundance was 3.26 × 10^6^ ± SE 3.29 × 10^5^ cells/mL, and bacterioplankton in aquarium seawater was 3.66 × 10^6^ ± SE 1.5 × 10^6^. For animals collected in the field, bacterial cell abundance in the coelomic fluid was 1.27 × 10^5^ ± SE 2.23 × 10^4^ cells/mL. Coelomocyte abundance was 2.73 × 10^5^ ± SE 6.74 × 10^4^ cells/mL, and bacterioplankton abundance in the tide pool was 8.95 × 10^5^ ± SE 1.57 × 10^5^. The low abundance of bacteria in the coelomic fluid was close to our detection limit of 9.77 × 10^3^ cells/mL.

## Discussion

Microscopic analysis of echinoderm tissue has established the widespread presence of gram-negative rods and spirilla bacterial cells in the subcuticular layer of echinoderms (de Souza Santos and da Silva Sasso, 1970; Holland and Nealson, 1978; Kelly et al., 1995; Kelly and McKenzie, 1995; Lawrence et al., 2010). These microbes have been hypothesized to be acquired early during the larval stage of development and likely exist as facultative residents due to the frequency of observed occurrence, typically 65% (Kelly et al., 1995; Kelly and McKenzie, 1995; Cerra et al., 1997). Indeed, the microbiome of sea star larvae appear to be similar to adults in taxonomic composition though a lower diversity was found among larvae which is likely due to a difference in sequencing depth as a result of differing sequencing methodologies (Galac et al., 2016). More recently, culture-independent approaches have been taken to characterize the microbial community associated with adult sea stars of *Acanthaster* cf. *solaris* (Crown-of-thorns star), *Asterias amurensis* (Japanese common star), and *Patiria pectinifera* (Blue bat star) (Nakagawa et al., 2017; Høj et al., 2018). Direct comparisons between studies is difficult because of varying sampling approaches, PCR protocols, and bioinformatics analyses used though broad similarities of microbial community composition found between studies do arise. The high relative abundance of *Helicobacter*-related taxon found previously in the coelomic fluid of *Asterias amurensis* was significantly less (<1% relative abundance) among the sea star taxa presented in this study (Nakagawa et al., 2017). Previous work documenting microbial communities associated with Crown-of-thorns sea star found four major bacterial groups driving tissue-specific patterns which included *Spirochaetales*, *Rhodobacterales*, *Oceanospirillales*, and *Mollicutes* (Høj et al., 2018). Our results corroborate these finding with the addition of *Chromatiales* and *Enterobacteriales* also making up observable differences between sample types (**Figures 6**, **7**). It appears that these microbial groups are largely driving tissue-specific patterns given the considerable overlap in low abundance sub-OTUs across all sample types (**Figure 7**). The factors driving these patterns in the microbial community composition in adults is not known, but likely is a combination of environmental factors (temperature, pH, salinity) and host factors (diet, mucosal layers, exposure to coelomocytes, secondary metabolites). It remains to be seen whether these statistical differences between body regions will translate to biologically meaningful differences.

The microbial community associated with sea stars appears to be distinct from seawater (**Figure 4B**). This has been reported for other benthic marine invertebrates including sea anemones, sea cucumbers, sponges, and corals (Ainsworth et al., 2015; Thomas et al., 2016; León-Palmero et al., 2018). If there is no selection or enrichment of microorganisms by sea stars, the microbial composition associated with sea stars would reflect that of their surrounding environment due to the water vascular system which functions by bringing water from their surrounding environment and distributing it through their body. Our results show that the microbial community associated with sea stars is significantly different from seawater with relatively few shared sub-OTUs despite the intake and circulation of seawater in the coelomic cavity (**Figure 3B**). The greater difference among shared sub-OTUs for animals collected in Washington likely is the result of the difference in depth between seawater sampled and animals collected. However, surface seawater was collected nearly at depth of animals in Queensland, Australia and still showed significantly different microbial community compositions. The microbiome of sea stars may be more influenced by the microbial composition of sediment, sediment pore water or more generally the benthic environment they live in. Geographic location was found to be a significant factor explaining the variance in community composition which could be the result of the different benthic environment these animals live in (**Figure 4B**). It is important to note that the primers used in this study are known to bias the representation of common marine bacterioplankton taxa in seawater (Apprill et al., 2015; Parada et al., 2016). Although it is uncertain whether these biases also extend to the microbial communities associated with sea stars, further studies are needed to fully evaluate the possibility.

It has been previously hypothesized that the bacterial community found in the coelomic fluid is transient and comes from allochthonous sources such as seawater (Nakagawa et al., 2017). The low observed abundance of bacteria we observed in the coelomic fluid of sea stars supports this hypothesis suggesting that the coelomic cavity may not contain a resident community of microorganisms (**Figure 8**). The subcuticular layer of sea stars appears to harbor a much greater abundance of bacteria estimated to be 10^8^–10^9^ g^−1^ ash-free dry wgt in comparison to the coelomic fluid while the density and abundance of bacteria in the gastrointestinal tract and gonads of sea stars remains unknown (Kelly et al., 1995; Kelly and McKenzie, 1995; Lawrence et al., 2010). A high abundance of bacteria in the coelomic fluid is not expected given the antimicrobial activity and phagocytic abilities of coelomocytes which likely prevent colonization of regions in the body cavity of healthy animals (Coteur et al., 2007; Pinsino et al., 2007; Smith et al., 2010). The coelomic fluid of sea stars is similar ionically and osmotically to their surrounding seawater but contains elevated potassium ions and small amounts of organic materials such as amino acids, sugars, and nitrogenous waste (Ferguson, 1964; Prusch and Whoriskey, 1976; Nakagawa et al., 2017). Despite the similarities between these environments, the bacterial abundance between the coelomic fluid and seawater ranged from one to two orders of magnitude in difference, signifying a strong selection against the presence of bacterial cells in the coelomic fluid (**Figure 8**). These differences were not as great for animals sampled in the field likely reflecting different physiological conditions as a product of their environment (natural vs artificial). Bacterial cells may be adventitious and coming into the coelomic cavity through the water vascular system or by leaking from tissues bacteria do colonize. Amplified DNA may then originate from live, dead or bacterial cells in the process of being phagocytized by the coelomocytes.

The importance, function, and influence the sea star microbiome has on the animal’s health and development is currently unknown. Recent functional predictions have been largely based on 16s rRNA community composition studies which are not a good predictor of community function. Nevertheless, current hypotheses regarding benefits provided by microbial symbionts include: sulfide detoxification, antifouling prevention, opportunistic pathogens, nitrogen fixation and nutrient acquisition (Lawrence et al., 2010; Nakagawa et al., 2017; Høj et al., 2018). Nitrogenase activity has been found in the gastrointestinal tract of sea urchins. Sea urchins are generally herbivores with low protein diets, and the presence of N_2_- fixing bacteria are thought to contribute to their total nitrogen demand. (Guerinot and Patriquin, 1981). Sea stars however generally do not have specialized diets, ingest structurally complex polysaccharides or require detoxification of dietary items all of which symbiotic microorganisms can assist with to improve animal nutrition (Douglas, 2009). None of the animals collected in this study have specialized diets though the sea stars from Queensland, Australia likely feed on biofilms to a greater extent than those collected in Washington, United States. The influence microorganisms might have on sea star nutrition or nutrient acquisition is not clear though this question has been experimentally approached. The nutritional intake of sea stars has been found to be a dual process of epidermal absorption of dissolved organic matter (DOM) from seawater and oral consumption (Bamford, 1982). The ability to absorb DOM through the epithelium is not unique to sea stars, but shared across many marine invertebrates (Gomme, 1982). Larvae and adult sea stars have the ability to selectively uptake neutral amino acids without the aid of bacteria, but the presence of bacteria within the sub-cuticular layer of sea stars might assist or compete for DOM (Manahan et al., 1983; Applebaum et al., 2013). Any nutritional benefit to the host might come from the host cropping the bacteria by phagocytosis though the line between nutritional benefit and sanitation in this situation is not clear. Further experimental testing is needed to investigate the impact of the microbiome to the animal’s nutritional economy and more generally the functional role of the sea star microbiome.

## Availability of Data and Material

The sequence data produced and analyzed for this study are available in the NCBI SRA repository under the accession number PRJNA383901. Script generated for analysis can be found at https://github.com/ewj34/Sea-Star-Microbiome.

## Author Contributions

EJ, IH, CP-R, SD, and DB designed and executed the research. EJ, IH, CP-R, and DB analyzed and interpreted the results. SD prepared samples for sequencing. All authors contributed to drafting of the manuscript.

## Conflict of Interest Statement

The authors declare that the research was conducted in the absence of any commercial or financial relationships that could be construed as a potential conflict of interest.
