# Supplementary material for: The Microbial Landscape of Sea Stars and the Anatomical and Interspecies Variability of Their Microbiome
Source: Front Microbiol. 2018 Aug 13;9:1829. doi: 10.3389/fmicb.2018.01829 (PMC6099117; doi:10.3389/fmicb.2018.01829)

Supplemental Figure 1– β diversity analysis of sea star and sea water microbial communities (**A**) UPGMA clustering based on Bray Curtis distances. Node tips are labeled by sea star taxa and node colors correspond to the respective order. Symbols correspond to sample type. Collapsed samples represent seawater samples (**B**) PCoA plots generated from Bray Curtis distances.

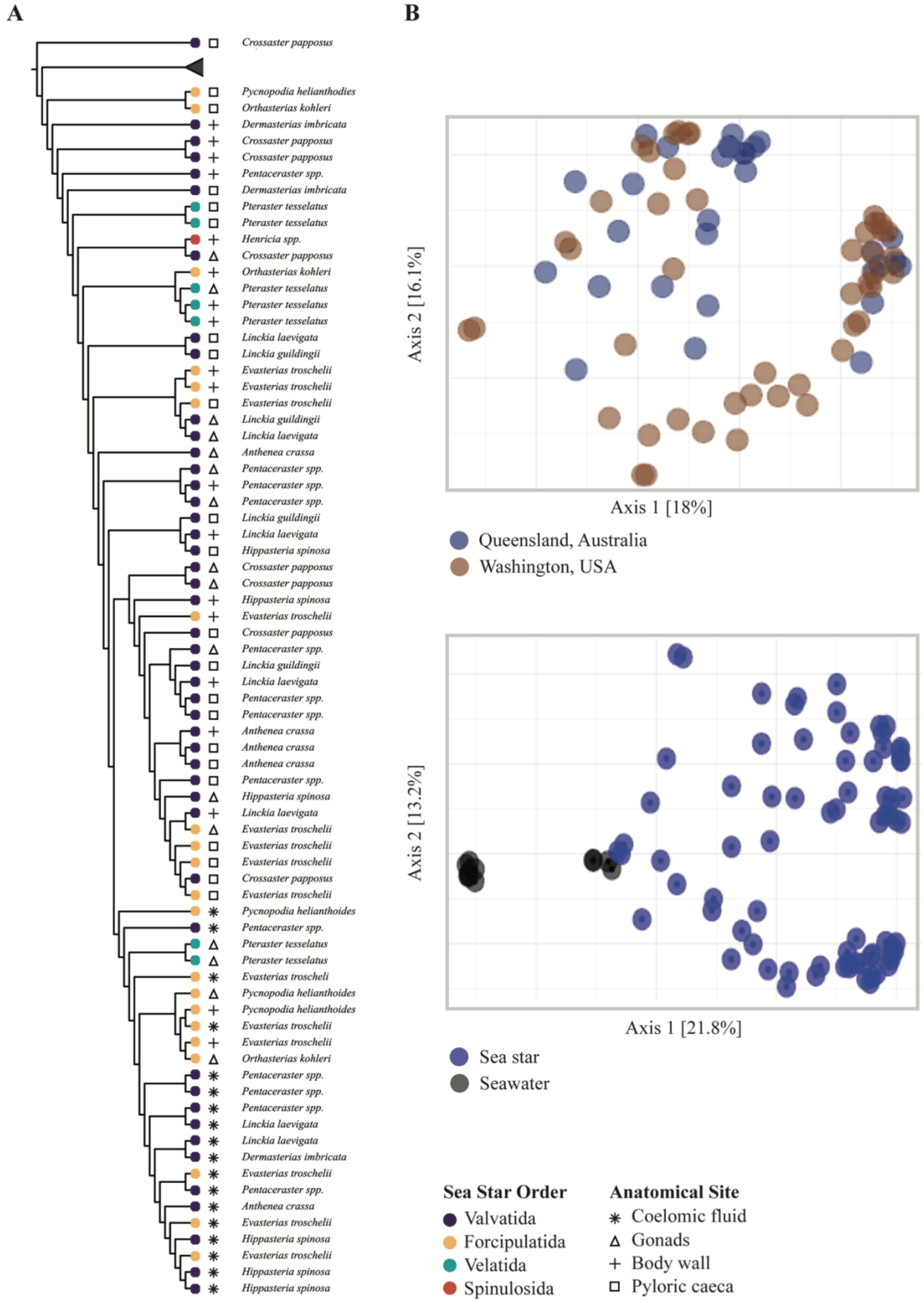

Supplement: Supplementary file 1 [file Image_1.PDF]
