# Supplementary material for: The Microbial Landscape of Sea Stars and the Anatomical and Interspecies Variability of Their Microbiome
Source: Front Microbiol. 2018 Aug 13;9:1829. doi: 10.3389/fmicb.2018.01829 (PMC6099117; doi:10.3389/fmicb.2018.01829)
Supplement: Supplementary file 2 [file Data_Sheet_1.PDF]

Supplementary Table 1 - Library metadata and read depth per library

Host.ID	location	Collection.Depth	GPS	Collection.Date	Sample.Amo	Host.Order	Host.Family	Genus.Species	Sample#	Sample	Sample.Type	TotalReadsPerLibrary
1	Moreton Bay, Australia	Subtidal	[-27.4802, 153.4034]	12_9_2015	0.1145 g	Valvatida	Oreasteridae	Anthenea crassa	X8106_PC	Pyloric Ceaca	PC	15022
2	Moreton Bay, Australia	Subtidal	[-27.4802, 153.4034]	12_10_2015	2 mL	Valvatida	Oreasteridae	Anthenea crassa	X8532_CF	Coelomic Fluid	CF	24620
2	Moreton Bay, Australia	Subtidal	[-27.4802, 153.4034]	12_10_2015	0.109 g	Valvatida	Oreasteridae	Anthenea crassa	X8533_TF	Epidermis (Tube Feet)	TF	4083
2	Moreton Bay, Australia	Subtidal	[-27.4802, 153.4034]	12_10_2015	0.1306 g	Valvatida	Oreasteridae	Anthenea crassa	X8534_G	Gonads	G	27518
2	Moreton Bay, Australia	Subtidal	[-27.4802, 153.4034]	12_10_2015	0.1013 g	Valvatida	Oreasteridae	Anthenea crassa	X8535_PC	Pyloric Ceaca	PC	6302
1	Moreton Bay, Australia	Surface	[-27.4802, 153.4034]	12_10_2015	1.2L	NA	NA	Australia Bacterioplankton	X8161_W	Sea Water	W	46581
2	Moreton Bay, Australia	Surface	[-27.4802, 153.4034]	12_10_2015	1.2L	NA	NA	Australia Bacterioplankton	X8163_W	Sea Water	W	20447
3	Heron Island, Australia	Surface	[-27.4802, 153.4034]	12_17_2015	6L	NA	NA	Australia Bacterioplankton	X8297_W	Sea Water	W	12928
4	Heron Island, Australia	Surface	[-27.4802, 153.4034]	12_17_2015	6L	NA	NA	Australia Bacterioplankton	X8298_W	Sea Water	W	2638
1	Puget Sound, Washington USA	35 m	[48.2934,-122.4296]	1_8_2016	0.1026 g	Valvatida	Solasteridae	Crossaster papposus	C324_G	Gonads	G	15961
1	Puget Sound, Washington USA	35 m	[48.2934,-122.4296]	1_8_2016	0.1027 g	Valvatida	Solasteridae	Crossaster papposus	C325_PC	Pyloric Ceaca	PC	31905
1	Puget Sound, Washington USA	35 m	[48.2934,-122.4296]	1_8_2016	0.1231 g	Valvatida	Solasteridae	Crossaster papposus	C326_TF	Epidermis (Tube Feet)	TF	23960
2	Puget Sound, Washington USA	37 m	[48.1133, -123.0648]	1_10_2016	0.0916 g	Valvatida	Solasteridae	Crossaster papposus	C563_PC	Pyloric Ceaca	PC	23425
2	Puget Sound, Washington USA	37 m	[48.1133, -123.0648]	1_10_2016	0.0977 g	Valvatida	Solasteridae	Crossaster papposus	C564_G	Gonads	G	10616
2	Puget Sound, Washington USA	37 m	[48.1133, -123.0648]	1_10_2016	0.0880 g	Valvatida	Solasteridae	Crossaster papposus	C565_TF	Epidermis (Tube Feet)	TF	17564
3	Puget Sound, Washington USA	50 m	[48.4228, -122.5716]	1_9_2016	0.1085 g	Valvatida	Solasteridae	Crossaster papposus	C446_PC	Pyloric Ceaca	PC	21774
3	Puget Sound, Washington USA	50 m	[48.4228, -122.5716]	1_9_2016	0.0997 g	Valvatida	Solasteridae	Crossaster papposus	C447_G	Gonads	G	34069
1	Puget Sound, Washington USA	28 m	[47.5589, -122.3553]	1_11_2016	2 mL	Valvatida	Asteropseidae	Dermasterias imbricata	C605_CF	Coelomic Fluid	CF	11606
1	Puget Sound, Washington USA	28 m	[47.5589, -122.3553]	1_11_2016	0.1152 g	Valvatida	Asteropseidae	Dermasterias imbricata	C606_PC	Pyloric Ceaca	PC	27980
1	Puget Sound, Washington USA	28 m	[47.5589, -122.3553]	1_11_2016	0.1245 g	Valvatida	Asteropseidae	Dermasterias imbricata	C607_TF	Epidermis (Tube Feet)	TF	22635
1	Puget Sound, Washington USA	30 m	[47.5810, -122.3339]	1_7_2016	2 mL	Forcipulatida	Asteriidae	Evasterias troschelii	C210_CF	Coelomic Fluid	CF	22736
2	Puget Sound, Washington USA	30 m	[48.2000, -122.5181]	1_10_2016	2 mL	Forcipulatida	Asteriidae	Evasterias troschelii	C528_CF	Coelomic Fluid	CF	2726
2	Puget Sound, Washington USA	30 m	[48.2000, -122.5181]	1_10_2016	0.0891 g	Forcipulatida	Asteriidae	Evasterias troschelii	C529_G	Gonads	G	2683
2	Puget Sound, Washington USA	30 m	[48.2000, -122.5181]	1_10_2016	0.0952 g	Forcipulatida	Asteriidae	Evasterias troschelii	C530_PC	Pyloric Ceaca	PC	14402
2	Puget Sound, Washington USA	30 m	[48.2000, -122.5181]	1_10_2016	0.0998 g	Forcipulatida	Asteriidae	Evasterias troschelii	C531_TF	Epidermis (Tube Feet)	TF	19155
3	Dutch Harbor, Alaska USA	Subtidal	[53.9,-166.53]	9_12_2015	2 mL	Forcipulatida	Asteriidae	Evasterias troschelii	T1EVA1_CF	Coelomic Fluid	CF	17335
3	Dutch Harbor, Alaska USA	Subtidal	[53.9,-166.53]	9_12_2015	0.0356 g	Forcipulatida	Asteriidae	Evasterias troschelii	T1EVA2_BP	Epidermis (Tube Feet)	TF	20357
3	Dutch Harbor, Alaska USA	Subtidal	[53.9,-166.53]	9_12_2015	0.0959 g	Forcipulatida	Asteriidae	Evasterias troschelii	T1EVA3_PC	Pyloric Ceaca	PC	36655
4	Dutch Harbor, Alaska USA	Subtidal	[53.9,-166.53]	9_12_2015	2 mL	Forcipulatida	Asteriidae	Evasterias troschelii	T1EVA4_CF	Coelomic Fluid	CF	26273
4	Dutch Harbor, Alaska USA	Subtidal	[53.9,-166.53]	9_12_2015	0.0343 g	Forcipulatida	Asteriidae	Evasterias troschelii	T1EVA5_BP	Epidermis (Tube Feet)	TF	53410
4	Dutch Harbor, Alaska USA	Subtidal	[53.9,-166.53]	9_12_2015	0.0911 g	Forcipulatida	Asteriidae	Evasterias troschelii	T1EVA6_PC	Pyloric Ceaca	PC	22569
5	Dutch Harbor, Alaska USA	Subtidal	[53.9,-166.53]	9_12_2015	2 mL	Forcipulatida	Asteriidae	Evasterias troschelii	T1EVA7_CF	Coelomic Fluid	CF	18860
5	Dutch Harbor, Alaska USA	Subtidal	[53.9,-166.53]	9_12_2015	0.0345 g	Forcipulatida	Asteriidae	Evasterias troschelii	T1EVA8_BP	Epidermis (Tube Feet)	TF	44363
5	Dutch Harbor, Alaska USA	Subtidal	[53.9,-166.53]	9_12_2015	0.1087 g	Forcipulatida	Asteriidae	Evasterias troschelii	T1EVA9_PC	Pyloric Ceaca	PC	23483
1	Puget Sound, Washington USA	30 m	[48.5810, -122.3339]	1_7_2016	0.1144 g	Spinulosida	Echinasteridae	Henricia spp.	C209_3_TF	Epidermis (Tube Feet)	TF	3710
1	Puget Sound, Washington USA	24 m	[47.3934, -122.2626]	1_6_2016	2 mL	Valvatida	Goniasteridae	Hippasteria spinosa	C7_CF	Coelomic Fluid	CF	24785
1	Puget Sound, Washington USA	24 m	[47.3934, -122.2626]	1_6_2016	0.1242 g	Valvatida	Goniasteridae	Hippasteria spinosa	C11_PC	Pyloric Ceaca	PC	19848
1	Puget Sound, Washington USA	24 m	[47.3934, -122.2626]	1_6_2016	0.1121 g	Valvatida	Goniasteridae	Hippasteria spinosa	C12_G	Gonads	G	14130
1	Puget Sound, Washington USA	24 m	[47.3934, -122.2626]	1_6_2016	0.1131 g	Valvatida	Goniasteridae	Hippasteria spinosa	C23_TF	Epidermis (Tube Feet)	TF	20150
2	Puget Sound, Washington USA	24 m	[47.3934, -122.2626]	1_6_2016	2 mL	Valvatida	Goniasteridae	Hippasteria spinosa	C10_CF	Coelomic Fluid	CF	28628
3	Puget Sound, Washington USA	24 m	[47.3934, -122.2626]	1_6_2016	2 mL	Valvatida	Goniasteridae	Hippasteria spinosa	C20_CF	Coelomic Fluid	CF	22589
1	Heron Island, Australia	Subtidal	[-23.4431, 151.9118]	12_17_2015	0.1053 g	Valvatida	Ophidiasteridae	Linckia guildingii	X8550_PC	Pyloric Ceaca	PC	6860
1	Heron Island, Australia	Subtidal	[-23.4431, 151.9118]	12_17_2015	0.1205 g	Valvatida	Ophidiasteridae	Linckia guildingii	X8551_G	Gonads	G	1215
2	Heron Island, Australia	Subtidal	[-23.4431, 151.9118]	12_19_2015	0.1225 g	Valvatida	Ophidiasteridae	Linckia guildingii	X8567_PC	Pyloric Ceaca	PC	33538
1	Heron Island, Australia	Subtidal	[-23.4431, 151.9118]	12_17_2015	2 mL	Valvatida	Ophidiasteridae	Linckia laevigata	X8540_CF	Coelomic Fluid	CF	34279
1	Heron Island, Australia	Subtidal	[-23.4431, 151.9118]	12_17_2015	0.0452 g	Valvatida	Ophidiasteridae	Linckia laevigata	X8541_TF	Epidermis (Tube Feet)	TF	8051
1	Heron Island, Australia	Subtidal	[-23.4431, 151.9118]	12_17_2015	0.1282 g	Valvatida	Ophidiasteridae	Linckia laevigata	X8543_PC	Pyloric Ceaca	PC	17972
2	Heron Island, Australia	Subtidal	[-23.4431, 151.9118]	12_18_2015	2 mL	Valvatida	Ophidiasteridae	Linckia laevigata	X8552_CF	Coelomic Fluid	CF	28676
2	Heron Island, Australia	Subtidal	[-23.4431, 151.9118]	12_18_2015	0.1007 g	Valvatida	Ophidiasteridae	Linckia laevigata	X8553_PC	Pyloric Ceaca	PC	50047
2	Heron Island, Australia	Subtidal	[-23.4431, 151.9118]	12_18_2015	0.0992 g	Valvatida	Ophidiasteridae	Linckia laevigata	X8554_TF	Epidermis (Tube Feet)	TF	2656
3	Heron Island, Australia	Subtidal	[-23.4431, 151.9118]	12_19_2015	0.1083 g	Valvatida	Ophidiasteridae	Linckia laevigata	X8557_TF	Epidermis (Tube Feet)	TF	33469
3	Heron Island, Australia	Subtidal	[-23.4431, 151.9118]	12_19_2015	0.1801 g	Valvatida	Ophidiasteridae	Linckia laevigata	X8558_G	Gonads	G	8127
1	Puget Sound, Washington USA	37 m	[48.1133, -123.0648]	1_10_2016	0.1032 g	Forcipulatida	Asteriidae	Orthasterias koehleri	C556_PC	Pyloric Ceaca	PC	38329
1	Puget Sound, Washington USA	37 m	[48.1133, -123.0648]	1_10_2016	0.1257 g	Forcipulatida	Asteriidae	Orthasterias koehleri	C557_G	Gonads	G	19589
1	Puget Sound, Washington USA	37 m	[48.1133, -123.0648]	1_10_2016	0.1221 g	Forcipulatida	Asteriidae	Orthasterias koehleri	C558_TF	Epidermis (Tube Feet)	TF	39991
1	Moreton Bay, Australia	Subtidal	[-27.4802, 153.4034]	12_10_2015	1 mL	Valvatida	Oreasteridae	Pentaceraster spp.	CF1_CF	Coelomic Fluid	CF	37736
2	Moreton Bay, Australia	Subtidal	[-27.4802, 153.4034]	12_10_2015	1 mL	Valvatida	Oreasteridae	Pentaceraster spp.	CF2_CF	Coelomic Fluid	CF	27580

3	Moreton Bay, Australia	Subtidal	[-27.4802, 153.4034]	12_10_2015	2 mL	Valvatida	Oreasteridae	Pentaceraster spp.	X8507_CF	Coelomic Fluid	CF	75964
3	Moreton Bay, Australia	Subtidal	[-27.4802, 153.4034]	12_10_2015	0.1078 g	Valvatida	Oreasteridae	Pentaceraster spp.	X8508_TF	Epidermis (Tube Feet)	TF	20498
3	Moreton Bay, Australia	Subtidal	[-27.4802, 153.4034]	12_10_2015	0.1069 g	Valvatida	Oreasteridae	Pentaceraster spp.	X8509_G	Gonads	G	21600
3	Moreton Bay, Australia	Subtidal	[-27.4802, 153.4034]	12_10_2015	0.1133 g	Valvatida	Oreasteridae	Pentaceraster spp.	X8510_PC	Pyloric Ceaca	PC	12792
4	Moreton Bay, Australia	Subtidal	[-27.4802, 153.4034]	12_10_2015	2 mL	Valvatida	Oreasteridae	Pentaceraster spp.	X8528_CF	Coelomic Fluid	CF	49399
4	Moreton Bay, Australia	Subtidal	[-27.4802, 153.4034]	12_10_2015	0.1118 g	Valvatida	Oreasteridae	Pentaceraster spp.	X8530_G	Gonads	G	20942
4	Moreton Bay, Australia	Subtidal	[-27.4802, 153.4034]	12_10_2015	0.1107 g	Valvatida	Oreasteridae	Pentaceraster spp.	X8531_PC	Pyloric Ceaca	PC	6938
5	Moreton Bay, Australia	Subtidal	[-27.4802, 153.4034]	12_10_2015	2 mL	Valvatida	Oreasteridae	Pentaceraster spp.	X8536_CF	Coelomic Fluid	CF	54647
5	Moreton Bay, Australia	Subtidal	[-27.4802, 153.4034]	12_10_2015	0.1083 g	Valvatida	Oreasteridae	Pentaceraster spp.	X8537_TF	Epidermis (Tube Feet)	TF	6972
5	Moreton Bay, Australia	Subtidal	[-27.4802, 153.4034]	12_10_2015	0.1247 g	Valvatida	Oreasteridae	Pentaceraster spp.	X8538_G	Gonads	G	34869
5	Moreton Bay, Australia	Subtidal	[-27.4802, 153.4034]	12_10_2015	0.1160 g	Valvatida	Oreasteridae	Pentaceraster spp.	X8539_PC	Pyloric Ceaca	PC	58076
1	Puget Sound, Washington USA	30 m	[48.3529, -122.5476]	1_8_2016	0.1053 g	Velatida	Pterasteridae	Pteraster tesselatus	C393_G	Gonads	G	5897
1	Puget Sound, Washington USA	30 m	[48.3529, -122.5476]	1_8_2016	0.0992 g	Velatida	Pterasteridae	Pteraster tesselatus	C394_PC	Pyloric Ceaca	PC	33023
1	Puget Sound, Washington USA	30 m	[48.3529, -122.5476]	1_8_2016	0.1192 g	Velatida	Pterasteridae	Pteraster tesselatus	C395_TF	Epidermis (Tube Feet)	TF	59682
2	Puget Sound, Washington USA	37 m	[48.1133, -123.0648]	1_10_2016	2 mL	Velatida	Pterasteridae	Pteraster tesselatus	C559_CF	Coelomic Fluid	CF	30003
2	Puget Sound, Washington USA	37 m	[48.1133, -123.0648]	1_10_2016	0.1509 g	Velatida	Pterasteridae	Pteraster tesselatus	C560_PC	Pyloric Ceaca	PC	160056
2	Puget Sound, Washington USA	37 m	[48.1133, -123.0648]	1_10_2016	0.1233 g	Velatida	Pterasteridae	Pteraster tesselatus	C561_G	Gonads	G	31433
2	Puget Sound, Washington USA	37 m	[48.1133, -123.0648]	1_10_2016	0.1276 h	Velatida	Pterasteridae	Pteraster tesselatus	C562_TF	Epidermis (Tube Feet)	TF	33854
1	Puget Sound, Washington USA	Surface	[47.5810, -122.3339]	1_7_2016	23L	NA	NA	Puget Sound Bacterioplankton	C227_W	Sea Water	W	98365
2	Puget Sound, Washington USA	Surface	[48.1412, -122.4779]	1_8_2016	21L	NA	NA	Puget Sound Bacterioplankton	C291_W	Sea Water	W	18243
3	Puget Sound, Washington USA	Surface	[48.2934,-122.4296]	1_8_2016	21L	NA	NA	Puget Sound Bacterioplankton	C322_W	Sea Water	W	12251
4	Puget Sound, Washington USA	Surface	[48.3529, -122.5476]	1_8_2016	21L	NA	NA	Puget Sound Bacterioplankton	C385_W	Sea Water	W	3309
5	Puget Sound, Washington USA	Surface	[48.4228, -122.5716]	1_9_2016	21L	NA	NA	Puget Sound Bacterioplankton	C445_W	Sea Water	W	21224
6	Puget Sound, Washington USA	Surface	[48.2000, -122.5181]	1_11_2016	21L	NA	NA	Puget Sound Bacterioplankton	C514_W	Sea Water	W	28569
7	Puget Sound, Washington USA	Surface	[48.1133, -123.0648]	1_11_2016	21 L	NA	NA	Puget Sound Bacterioplankton	C554_W	Sea Water	W	16520
1	Puget Sound, Washington USA	30 m	[48.2000, -122.5181]	1_10_2016	2 mL	Forcipulatida	Asteriidae	Pycnopodia helianthoides	C511_CF	Coelomic Fluid	CF	2250
1	Puget Sound, Washington USA	30 m	[48.2000, -122.5181]	1_10_2016	0.1195 g	Forcipulatida	Asteriidae	Pycnopodia helianthoides	C512_PC	Pyloric Ceaca	PC	38053
1	Puget Sound, Washington USA	30 m	[48.2000, -122.5181]	1_10_2016	0.1052 g	Forcipulatida	Asteriidae	Pycnopodia helianthoides	C515_G	Gonads	G	12212
1	Puget Sound, Washington USA	30 m	[48.2000, -122.5181]	1_10_2016	0.0996 g	Forcipulatida	Asteriidae	Pycnopodia helianthoides	C516_TF	Epidermis (Tube Feet)	TF	13231
